# A comparative evaluation of microleakage of three different newer direct composite resins using a self etching primer in class V cavities: An *in vitro* study

**DOI:** 10.4103/0972-0707.58340

**Published:** 2009

**Authors:** Mithra N Hegde, Pallavi Vyapaka, Shishir Shetty

**Affiliations:** Department of Conservative and Endodontics, A. B. Shetty Memorial Institute of Dental Sciences, India

**Keywords:** Class V cavities, microleakage, nanocomposites, nanoceramic composites, nanohybrid composites, self-etch adhesive

## Abstract

**Aims/Objectives:**

The aim of this *in vitro* study is to study, measure and compare the microleakage in three different newer direct composite resins using a self-etch adhesive bonding system in class V cavities by fluorescent dye penetration technique.

**Materials and Methods:**

Class V cavities were prepared on 45 human maxillary premolar teeth. On all specimens, one coat of G-Bond (GC Japan) applied and light cured. Teeth are then equally divided into 3 groups of 15 samples each. Filtek Z350 (3M ESPE), Ceram X duo (Dentsply Asia) and Synergy D6 (Coltene/Whaledent) resin composites were placed on samples of Groups I, II and III, respectively, in increments and light cured. After polishing the restorations, the specimens were suspended in Rhodamine 6G fluorescent dye for 48 h. The teeth were then sectioned longitudinally and observed for the extent of microleakage under the florescent microscope.

**Statistical Analysis Used:**

The results were subjected to statistical analysis using Kruskal Wallis and Mann–Whitney U Test.

**Results:**

Results showed no statistically significant difference among three groups tested.

**Conclusions:**

None of the materials tested was able to completely eliminate the microleakage in class V cavities.

## INTRODUCTION

Dentistry had always thrived to achieve biocompatible restorations that do not compromise the pulp and also maintain the dental seal. One of the significant contributions has been the development of resin- based composite technology.[1]

The major disadvantage of visible light-cured composites is polymerization shrinkage. This shrinkage can result in a gap formation between the composite material and tooth structure, particularly if the restoration margin is placed in dentin or cementum.[2] Bacteria, fluids, molecules, or ions can pass through this gap between the resin composite and the cavity wall, a process called microleakage.[3] Microleakage is thought to be responsible for hypersensitivity, secondary caries, pulpal pathosis and failure of restorations.[4] Besides pulpal irritation and secondary caries, microleakage also results in marginal discoloration.

Direct class V restorations can be placed to an acceptable standard if the gingival margin is on sound enamel; however, the quality of the marginal sealing of adhesive restorations located below the cemento-enamel junction is still questionable.[5]

Therefore, the purpose of this investigation is to study the microleakage of class V cavities restored with three different newer direct composite materials using a self-etching adhesive by fluorescent dye penetration method.

## MATERIALS AND METHODS

Forty-five extracted intact human maxillary premolars were collected, stored, disinfected and handled as per the recommendations and guidelines laid down by OSHA and CDC.

Class V cavity preparation with incisal margins in enamel and gingival margins in cementum or dentin were performed on the labial surface of each tooth. Preparations were centered on the CEJ and had the following dimensions: 2 mm axial depth, 3 mm occlusogingival height and 5 mm buccolingual width. Margins received 45° bevel width of 0.5-1 mm.

One coat of VII generation bonding agent G-Bond (GC Japan) was applied to all the samples and left undisturbed for 10 s, after which strong air drying was performed for 5 s. Subsequently, light curing of the surface was done for 10 s. The samples were randomly and equally divided into 3 groups of 15 samples each. All samples were restored with composite resins as per the grouping.

Group I – Restored with Filtek Z350 (3M ESPE) nanocomposite material.

Group II – Restored with Ceram X duo (Dentsply Asia), nanoceramic composite material.

Group III – Restored with Synergy D6 (Coltene/Whaledent) nanohybrid composite material.

Then all the groups were light cured for 40 s by using QHL75 (light curing unit).

Each restoration was finished with So-Flex finishing discs operated at high speed using a water coolant.

### Dye leakage

After the placement of the restorations, the teeth were stored in 37°C water, except when they were removed from storage and subjected to 250 thermal cycles between 5°C and 55°C water baths. Dwell time was 1 min with a 10-s transit time between baths. The samples were painted with nail polish leaving 1 mm around the restoration. The apices were sealed with sticky wax 200 ml of Rhodamine 6G fluorescent dye was prepared with the dilution of 1:10 and placed in 45 vials, each containing 15 ml of the dye. The samples were suspended in these vials for 48 h. Following this, the samples were washed with water and the nail polish was removed. The teeth were then sectioned longitudinally using carborundum disks and thin sections were prepared, which were placed on slides and observed under the microscope. The assessment of dye penetration when viewed under the fluorescent microscopy was scored[6] by an investigator blindly at all interfaces. The most severe degree of dye penetration was recorded for each section. The dye penetration for composite/tooth interface was scored for occlusal/cervical margins on a non-parametric scale from 0 to 4 based on the ordinal ranking system, and the degree of leakage on the enamel and dentinal/cemental margins were determined.

### Scoring criteria

**Table:** 

0	No dye leakage
1	Dye leakage up on one-third the cavity wall.
2	Dye leakage up to two-third the cavity wall
3	Dye leakage along the last one-third and up to the axial wall
4	Dye leakage along the whole of the axial wall.

The data obtained from the present study were statistically analyzed by Kruskal-Wallis and Mann–Whitney U test. The software used for analyzing the data was “SPSS version 15-O.”

## RESULTS

When the data was subjected to statistical analysis, the following results were obtained:

The comparison of mean percentage leakage at the enamel and cemental/dentinal margin among the three groups showed a statistically insignificant result; however, experimentally significant values with least leakage at enamel margins was obtained for Group I and a greater degree of leakage was observed in Group II [Table 1].

**Table 1:** Comparison of values of mean percentage leakage at the enamel and dentinal margins among three groups by Kruskal Wallis test

	*N*	Mean rank at the enamel margins	Mean rank at the dentinal margins
Group I	15	21.13	21.23
Group II	15	24.47	24.77
Group III	15	23.40	23.00
Total mean value		1.84	2.31
*P* value		0.768	0.580

*P* = Probability (<0.05 level of significance)

However, experimentally at the enamel and dentinal/cemental restoration interface, Group II showed greater leakage values and Group I showed the least mean leakage values.

## DISCUSSION

Recent advances in dental adhesives as well as an increased demand for esthetics have stimulated a great increase in the use of resin composites. Despite the remarkable developments in the technology of the resin composite restorative materials, clinical failures of resin restorations are still reported.

This study investigated the microleakage of Ceram X duo (Dentsply), Filtek Z350 (3M ESPE) and SynergyD6 (Coltene/Whaledent) when used in class V restorations.

Polymerization shrinkage remains a major disadvantage for composite restorations. Shrinkage produces stress at the adhesive interface, which could lead to bonding failure with gap formation. The stress generated could reach up to 10 MPA, leading to a marginal breakdown.[7] Microleakage evaluation is the most common method of assessing the sealing efficiency of a restorative material.[8] This shrinkage stress depends on factors such as cavity size and shape, substrate type and location of the margins, restorative material and technique of placement and polymerization.[9]

The class V lesion presents special problems with any restorative material because the selected material is required to bond to enamel and dentin/cementum. Dentin is a less favorable substrate than enamel for resin bonding. It was difficult to obtain good adhesion to dentine or cementum. In the present study, no material could completely eliminate microleakage at the dentine or cementum margin.

The various nanofilled composites that were used in this study have higher filler loading compared to conventional resin composites. Filtek Z350 (3M ESPE) Universal Restorative is a nanocomposite that contains a combination of a nonagglomerated/nonaggregated, 20 nm nanosilica fillers, and loosely bound agglomerated zirconia/silica nanocluster, consisting of agglomerates of primary zirconia/silica particles with 5-20 nm fillers. The cluster particle size range is from 0.6 to 1.4 microns.[10] Ceram X duo comprises organically modified ceramic nanoparticles and nanofillers combined with conventional glass fillers of approximately 1 μm. The size of the nanoceramic particles was found to be approximately 2.3 nm.[11] The Synergy D6 (Colténe/Whaledent) is a nanohybrid, composite, which incorporates spherical nanosized fillers mainly as nanoadditives, also known as rheological modifiers in the form of nanocolor pigments. The average filler particle size is 0.6 μm.[12]

The dentine bonding agent used in this study is G-Bond (GC Japan), which is a one-step, self-etch dentine bonding agent that forms a nonconventional interface with the dentin – a “Nano Interaction Zone” (NIZ) with minimal decalcification and almost no exposure to collagen fibers. This “nano” level reaction produces an insoluble calcium compound for a better bond that is less likely to deteriorate from enzymes present in the mouth.[13]

The results of the present study demonstrate better sealing ability in enamel than in dentin or cementum margins, and this is in accordance with the previous findings.[6,14,15] It was found that no material could completely eliminate microleakage at the enamel margin. Group I showed better results in comparison to Groups II and III.

Possible reasons for microleakage of contaminants at the dentin restoration margin includes the following: Cavity configuration (C- factor), dentinal tubule orientation to the cervical wall (CEJ), organic content of dentine substrate and movement of dentinal tubular fluids, incomplete alteration or removal of smear layer by acidic primers (self- etch system) for adequate demineralization and hybrid layer formation, inefficient infiltration/ penetration of primer components into the demineralized collagen fibrils, dentin substrates hydration level (solvent carries in the adhesive agent reacting differently with varying degrees of surface moisture), incomplete evaporation of the solvent from the dentin surface prior to attachment of the adhesive monomers, incompatibility of the bonding agent used in this study with the respective resin composite, acid component composition (pH, osmolarity, thickening agent), polymerization contraction, physical characteristics of the restorative material, (filler loading, volumetric expansion, modulus of elasticity), inadequate margin adaptation of restorative material, polymerization source-photoinitiator incompatibilities and instrumentation, and finishing and polishing effects.

Possible reasons of microleakage could vary from specimen to specimen. However, the results of this study demonstrate that the dynamic nature of substrate morphology is indeed an important factor and possibly the reason for adhesion defects of the restorative materials to the tooth structure.

## CONCLUSION

Under the limitations of the present study, none of the tested materials was able to eliminate microleakage at enamel or dentinal/cemental margins in a class V cavity preparation and there is no statistically significant difference in microleakage among the three groups tested. However, an experimentally improved result was obtained for Group I at both the enamel and dentinal/cemental margins. Clinical trials should be performed to assess the performance of these composite restorative materials before definitive conclusions are formulated.

## References

[1] Mitra SB, Wu D, Holmes BN (2003). An application of nanotechnology in advanced dental materials. J Am Dent Assoc.

[2] Yazici AR, Celik C, Ozgünaltay G (2004). Microleakage of different resin composite types. Quintessence Int.

[3] Hilton TJ, Schwartz RS, Ferracane JL (1997). Microleakage of four class II resin composite insertion techniques at intraoral temperature. Quintessence Int.

[4] Franco EB, Gonzaga Lopes L, Lia Mondelli RF, da Silva e Souza MH, Pereira Lauris JR (2003). Effect of the cavity configuration factor on the marginal microleakage of esthetic restorative materials. Am J Dent.

[5] Loguercio AD, de Oliveira Bauer JR, Reis A, Grande RH (2004). *In vitro* microleakage of packable composites in Class II restorations. Quintessence Int.

[6] Alavi AA, Kianimanesh N (2002). Microleakage of direct and indirect composite restorations with three dentine bonding agents. Oper Dent.

[7] Duquia Rde C, Osinaga PW, Demarco FF, de V Habekost L, Conceição EN (2006). Cervical microleakage in MOD restorations: *In vitro* comparison of indirect and direct composite. Oper Dent.

[8] Yamazaki PC, Bedran-Russo AK, Pereira PN, Wsift EJ (2006). Microleakage evaluation of a new low-shrinkage composite restorative material. Oper Dent.

[9] Araujo Fde O, Vieira LC, Monteiro Junior S (2006). Influence of resin composite shade and location of the gingival margin on the microleakage of posterior restorations. Oper Dent.

[10] http://www.delhidentist.in/FiltekZ350.pdf.

[11] http://www.dentsplymea.com/CeramX_sc.pdf.

[12] http://www.coltenewhaledent.com.

[13] http://www.gcamerica.com/gcgbond.html.

[14] Castelnuovo J, Tjan AH, Liu P (1996). Microleakage of multi-step and simplified- step bonding systems. Am J Dent.

[15] Saunders WP, Saunders EM (1996). Microleakage of bonding agents with wet and dry bonding techniques. Am J Dent.

